# SCFAs strongly stimulate PYY production in human enteroendocrine cells

**DOI:** 10.1038/s41598-017-18259-0

**Published:** 2018-01-08

**Authors:** P. Larraufie, C. Martin-Gallausiaux, N. Lapaque, J. Dore, F. M. Gribble, F. Reimann, H. M. Blottiere

**Affiliations:** 1 University of Cambridge, Metabolic Research Laboratories and MRC Metabolic Diseases Unit, WT-MRC Institute of Metabolic Science, Addenbrooke’s Hospital, Cambridge, UK; 2 Micalis Institute, INRA, AgroParisTech, Université Paris-Saclay, 78350 Jouy-en-Josas, France; 3 Sorbonne Universités, UPMC Univ Paris 06, IFD, 4 place Jussieu, 75252 Paris, cedex 05 France; 4 US 1367 MetaGenoPolis, INRA, Université Paris-Saclay, 78350 Jouy en Josas, France

## Abstract

Peptide-YY (PYY) and Glucagon-Like Peptide-1 (GLP-1) play important roles in the regulation of food intake and insulin secretion, and are of translational interest in the field of obesity and diabetes. PYY production is highest in enteroendocrine cells located in the distal intestine, mirroring the sites where high concentrations of short chain fatty acids (SCFAs) are produced by gut microbiota. We show here that propionate and butyrate strongly increased expression of *PYY* but not *GCG* in human cell line and intestinal primary culture models. The effect was predominantly attributable to the histone deacetylase inhibitory activity of SCFA and minor, but significant contributions of FFA2 (GPR43). Consistent with the SCFA-dependent elevation of *PYY* gene expression, we also observed increased basal and stimulated PYY hormone secretion. Interestingly, the transcriptional stimulation of *PYY* was specific to human-derived cell models and not reproduced in murine primary cultures. This is likely due to substantial differences in *PYY* gene structure between mouse and human. In summary, this study revealed a strong regulation of PYY production by SCFA that was evident in humans but not mice, and suggests that high fibre diets elevate plasma concentrations of the anorexigenic hormone PYY, both by targeting gene expression and hormone secretion.

## Introduction

The gut represents one the most important endocrine organs, secreting more than 20 different hormones from a scarce and heterogeneous enteroendocrine cell (EEC) population. Among these hormones, Peptide-YY (PYY) and Glucagon-like-peptide-1 (GLP-1) are of specific interest in the field of obesity and diabetes as they have been implicated in the regulation of food intake and insulin secretion, even though their precise physiological significance in healthy and pathological states is still debated^1–6^. Moreover, these two peptides are co-secreted from the same subpopulation of EECs, commonly referred to as L-cells^7^.

Many studies have focused on the regulation of secretion by these cells, but studies into the mechanisms controlling the production of the prohormones have been limited^7,8^. In humans, PYY is mainly produced in the colon, where short chain fatty acids (SCFAs) are produced at high levels through the fermentation of fibre by gut microbiota^9^. SCFAs exert a number of actions on host cells, as they serve as energy substrates, signalling molecules through specific G-protein coupled receptors FFA2, FFA3 and GPR109a, as well as intracellular signalling molecules inhibiting Lysine/Histone Deacetylase (HDACs)^10–13^. Human FFA2 and FFA3 have slightly different preferences for SCFAs. Both receptors have affinities in the order of 0.1 mM for propionate and butyrate, whereas for acetate the affinities are ~0.1 mM for FFA2 and >1 mM for FFA3^14,15^. HDAC inhibition by butyrate is known to regulate the expression of a number of genes in different tissues and cell lines, and its function is mainly dependent on SP1/SP3 binding sites and independent of surface membrane SCFA receptors^16–18^.

High SCFA concentrations have been associated with elevated plasma levels of PYY and GLP-1 in humans and mice, mainly after consumption of digestion-resistant fibre diets resulting in increased colonic fermentation^19–21^. Two main mechanisms have been described to date: an acute effect of SCFAs on hormone secretion through activation of the Gq coupled receptor FFA2^22–24^; and an increased number of EECs, particularly GLP-1 and PYY expressing cells^25–27^. Zhou and collaborators also proposed that SCFAs may directly regulate *Pyy* and *Gcg* (encoding Proglucagon) gene expression in rats, but limited their experiments to short periods of time and did not take into account a potential increased number of EECs resulting in apparent increased tissue content^28^. Most of the functional studies were performed in rodent models, while validation in humans is still lacking or did only observe acute stimulation at high, possibly supraphysiological, concentrations^29^. This may be explained by the differences in receptor expression between human and mouse L-cells, as human L-cells express much lower levels of *FFAR3* than *FFAR2* ^30^, or by the different affinities of the two receptors for SCFAs. Compared with their human counterparts, mouse FFA2 has a lower affinity for SCFAs by 0.5–1 orders of magnitude whereas mouse FFA3 has a higher affinity for acetate and propionate, but similar affinity for butyrate^15^. These differences are not predicted to affect SCFA sensing in the colon where the concentrations are several orders of magnitude higher, but could explain differences in the detection of circulating SCFAs which are in the 10–100 μM range^15^. However, as SCFAs seem capable to alter PYY and GLP-1 plasma levels in humans^29^, other mechanisms may be involved and need to be assessed.

## Results

### NCI-h716 and HuTu-80 as EEC models for assessing SCFA responses

**Figure 1:**
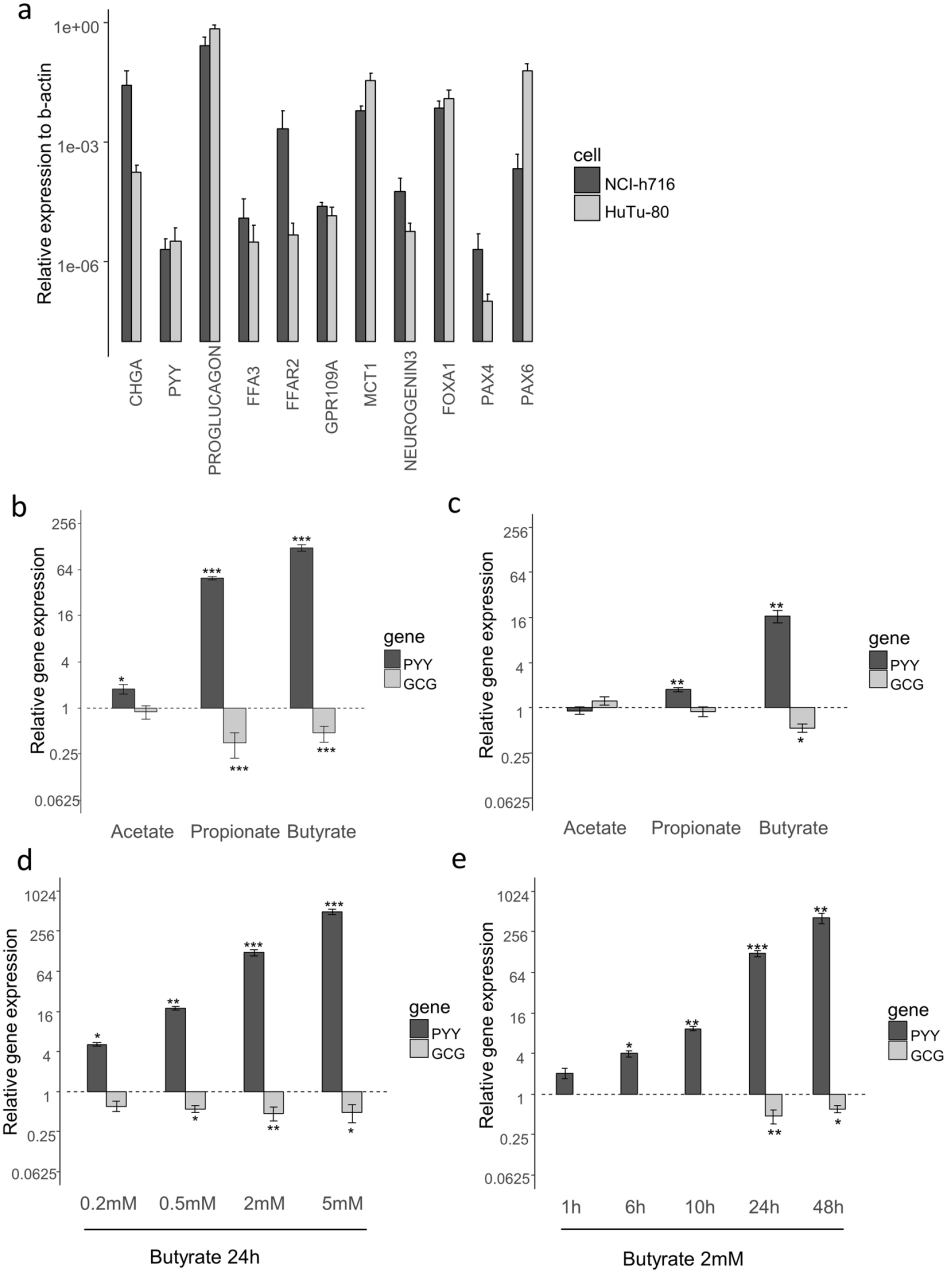
SCFAs increase *PYY* expression in human EEC model cell lines. (**a**) Expression of EEC markers, SCFA transporters and receptors and specific enteroendocrine differentiation factors in NCI-h716 (dark grey) and HuTu-80 (light grey) were detected by RT-qPCR. Data are expressed relative to *β-actin* determined by the 2^−ΔCt^ method, on at least 4 distinct experiments performed in duplicate, represented as means ± s.e.m. N.D.: Not Detected. (**b**,**c**) Relative expression to control of *PYY* (dark grey) and *GCG* (light grey) after treatment with SCFAs (2 mM, 24 h) on NCI-h716 cells (**b**) and HuTu-80 cells (**c**). (**d**,**e**) Effect of concentration (**d**) or time of incubation (**e**) of butyrate on *PYY* expression in NCI-h716 cells. *PYY* expression relative to control expression is determined by the 2^−ΔΔCt^ method using *β-actin* as control gene. Data are means ± s.e.m. of at least three distinct experiments. (***P < 0.001; **P < 0.01; *P < 0.05, Dunn to control test).

Studies on human EECs have been limited due to the scarcity of the cellular population, the absence of external markers and the limited models available. We used two cell lines modelling human GLP-1 secreting EECs, namely NCI-h716 and HuTu-80, derived respectively from a human cecum adenocarcinoma and a duodenum adenocarcinoma^31,32^, to assess effects of SCFAs on prohormone gene expression. We validated the expression of different enteroendocrine markers in these cell lines, and confirmed the expression of *PYY* and *GCG* (*PROGLUCAGON*) in both cell lines, even though *PYY* expression was low (Fig. 1a). These two cell lines also expressed different levels of SCFA receptors and transporters, with the NCI-h716 cell line being the most similar to colonic GLP1-expressing-cells with a higher expression of *FFAR2*.

### Effect of SCFAs on *PYY* and *GCG* gene expression

Incubation of NCI-h716 cells with SCFAs (each at a concentration of 2 mM, which is above the EC50 of both receptors and known to induce HDAC inhibition in different models) for 24 h increased *PYY* expression to different extents depending on the SCFA chain length (Fig. 1b). Butyrate was the most effective activator, increasing gene expression ~120-fold. Propionate had a smaller but still very strong effect, increasing expression ~40-fold, whereas the effect of acetate was more modest, increasing expression only ~2-fold. This effect was specific for *PYY* as propionate and butyrate slightly decreased *GCG* expression. Moreover, the effect of butyrate was time and dose dependent; we could not reach saturation in the dose or time range, due to negative effects on cell viability with more prolonged incubations or elevated concentrations (Fig. 1d,e). HuTu-80 cells yielded qualitatively similar results for *PYY*-specific transcriptional up-regulation, although compared with NCI-h716 cells the fold increases in *PYY* expression were lower for butyrate (~16-fold increase) and propionate (~2-fold increase), and no significant change was observed with acetate (Fig. 1c).

### Mechanisms involved in the effects of SCFAs

**Figure 2:**
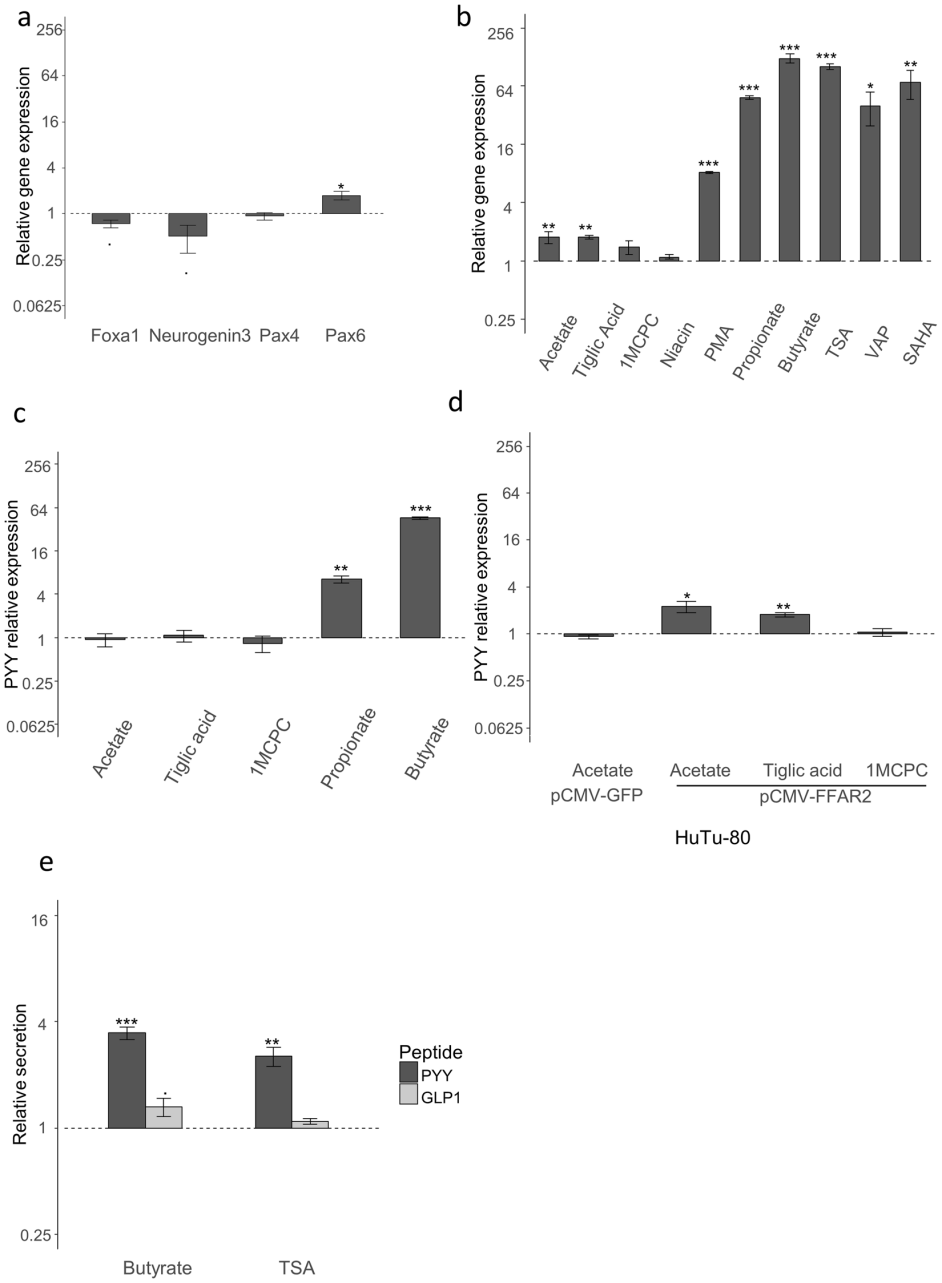
Mechanisms involved in response to SCFAs. (**a**) Expression relative to control of PYY-producing EEC characteristic transcription factors after incubation with 2 mM butyrate for 24 h in NCI-h716 cells. (**b**) Effect of 24 h incubation with SCFAs, receptor agonists, tiglic acid (FFA2, 100 μM), 1-MCPC (FFA3, 100 μM) and niacin (GPR109a, 1 mM), PMA (200 nM), TSA (500 nM), Valproic acid (5 mM) or SAHA (5 μM) on *PYY* expression in NCI-h716 cells. (**c**) Effect of 24 h incubation with SCFAs and receptor agonists on *PYY* expression in FFA2 depleted NCI-h716 cells. (**d**) Expression of *PYY* relative to untreated cells in response to acetate (2 mM), tiglic acid (100 μM) or 1MCPC (100 μM) in Hutu-80 cells transfected with pCMV-FFAR2 or pCMV-eGFP. (**e**) Secretion relative to control of PYY (dark grey) or GLP-1 (light grey) by NCI-h716 pre-incubated with butyrate (2 mM) or TSA (500 nM) for 24 h, and subsequently incubated in non-stimulated conditions for 2 h; measured by ELISA. Mean basal secretion was 15.6 ± 1.6 pg/mL for PYY and 90.6 ± 4.3 pg/mL or GLP-1. Data are means ± s.e.m. of four distinct experiments. (***P < 0.001; **P < 0.01; *P < 0.05, Dunn to control test).

*In vivo*, SCFAs have been shown to alter EEC differentiation and to increase their number. We therefore tested whether the effect of butyrate could be explained by a change of transcription factor expression. We could not detect any significant change in the expression of *NEUROGENIN3*, *FOXA1, PAX4 or PAX6*, which have previously been implicated in EEC differentiation towards *GCG* expression^25,26^ (Fig. 2a).

Human cells could respond to SCFAs at the concentration tested through two main mechanisms: activation of specific G-protein coupled receptors (FFA2, FFA3 and GPR109a) and inhibition of histone deacetylases (HDACs). To test the roles of these receptors, we first used selective agonists. Only the FFA2 agonist, tiglic acid, but not FFA3 or GPR109a agonists (1-methylcyclopropane carboxylate (1-MCPC) and niacin, respectively), induced a significant increase of *PYY* expression, which was of similar magnitude to acetate (Fig. 2b). Activation by PMA of PKC, which is downstream in the Gq pathway, also enhanced *PYY* expression, supporting the potential involvement of the Gq/PKC pathway in the response to FFA2 activation, in line with this pathway also mediating the acute effects of FFA2 activation on GLP-1 secretion^33^.To further investigate the role of FFA2 in NCI-h716 cells, we depleted the receptor using Crispr/Cas9. Depletion of FFA2 was validated at the functional level, and we also confirmed that FFA2 depletion did not alter the *FFAR3* gene sequence or its expression (Supplementary Figure 1). In FFA2-depleted cells, acetate or receptor agonists no longer induced any increase of *PYY* expression, whereas propionate and butyrate still induced strong responses, although diminished approximately 2-fold compared with control cells (Fig. 2c). Interestingly, acetate did not induce any effect on *PYY* expression in HuTu-80 cells, which endogenously express relatively low *FFAR2* levels, but over-expression of *FFAR2* was sufficient to induce increased *PYY* expression after acetate incubation, confirming a role for FFA2 in the response to acetate (Fig. 2d). We therefore concluded that the main effect of butyrate and propionate is not due to activation of the known G-protein coupled SCFA receptors.

We then used Trichostatin A (TSA), suberoylanilide hydroxamic acid (SAHA) and valproic acid (VPA), which like butyrate and propionate, inhibit class I and II HDACs^16,34,35^. All three agents induced a similar strong effect on *PYY* expression in NCI-h716 cells (Fig. 2b). These results suggested that K/HDAC inhibition, an extensively described GPCR-independent activity of butyrate and propionate, underlies the observed strong increase of *PYY* expression. Taken together, these results suggested that SCFAs modulated *PYY* expression by two different but additive pathways: stimulation of FFA2 by all three SCFAs induced a small increase in *PYY* mRNA levels, whereas HDAC inhibition by propionate and butyrate induced a very large increase in *PYY* mRNA levels.

Increased *PYY* gene expression after butyrate or TSA treatment translated into elevated PYY secretion. Compared to controls, NCI-h716 cells incubated for 24 h with butyrate or TSA secreted four times more PYY when subsequently incubated for 2 h in a buffer lacking secretory stimuli. By contrast, GLP-1 secretion was unchanged by the preincubations, mirroring the results found at the gene expression level (Fig. 2e).

### Effect is specific to human tissues

**Figure 3:**
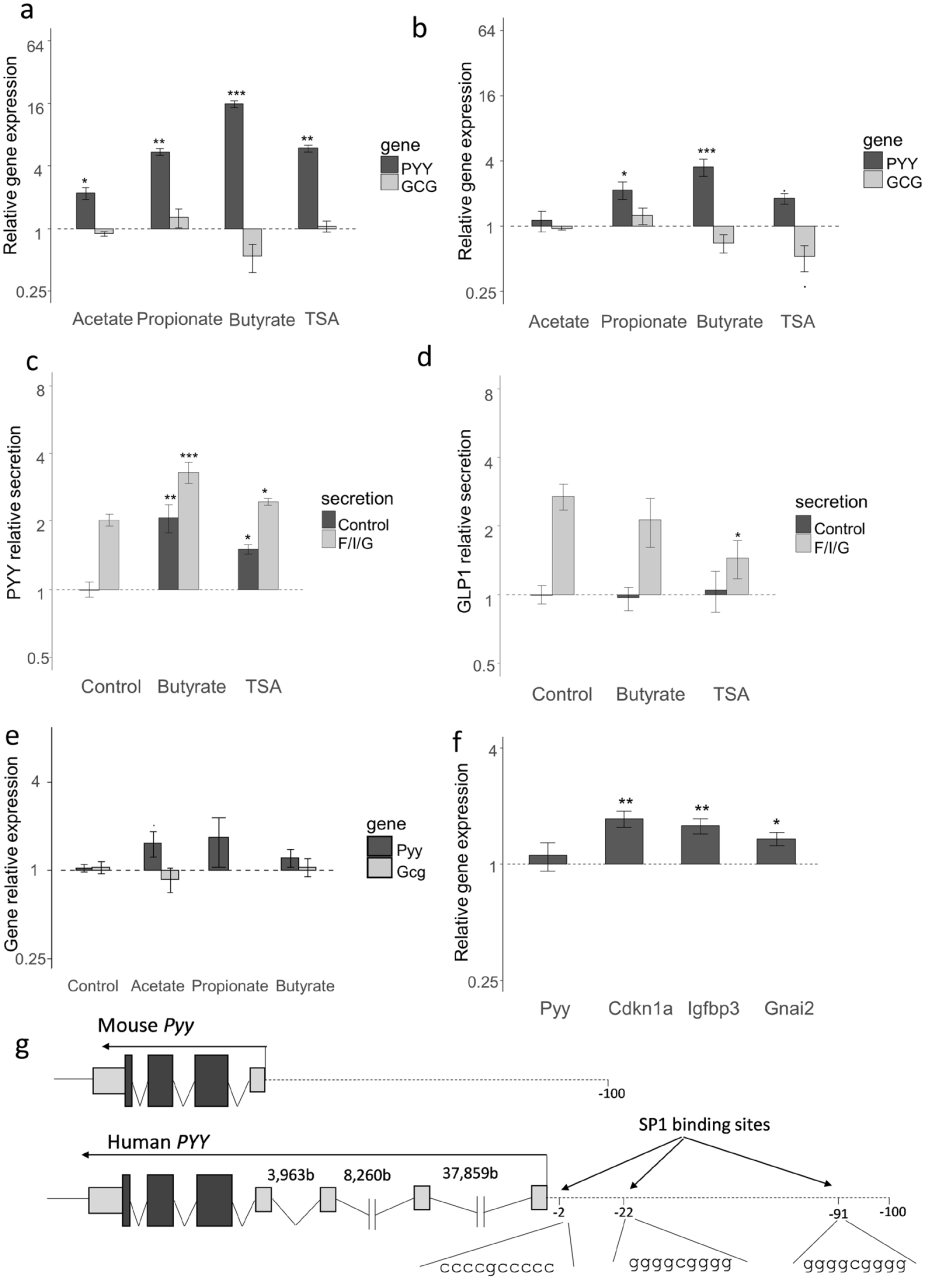
Effect of SCFAs is species dependent. (**a**,**b**) Expression relative to untreated control cultures of *PYY* (dark grey) and *GCG* (light grey) in human colonic (**a**) and duodenal (**b**) cultures after incubation with SCFAs (2 mM) or TSA (500 nM). (**c**,**d**) Secretion, relative to control, of PYY (**c**) or GLP-1 (**d**) by colonic primary cultures pre-incubated with butyrate (2 mM) or TSA (500 nM) for 24 h, and subsequently incubated for 2 h in the absence of stimuli (dark grey) or in the presence of forskolin (10 μM), IBMX (10 μM) and glucose (10 mM) (light grey); measured by ELISA. Secretion is normalized to the protein content in the lysed cells, measured by BCA assay. Mean basal secretion was 0.42 pg/mL/μg of tissue and 0.019 pg/mL/μg of tissue for PYY and GLP-1, respectively. Analysis was performed comparing cells incubated with either butyrate or TSA to control-incubated cells, in both secretion conditions separately (***P < 0.001; **P < 0.01; *P < 0.05, Dunn to control test). (**e**) Expression of *Pyy* and *Gcg* in primary mouse colonic cultures after incubation with SCFAs (2 mM), relative to control-treated cultures. Data are means ± s.e.m. of four distinct experiments. (**f**) Effect of TSA (500 nM, 24 h) on the expression of genes known to be regulated by HDAC inhibition and on *Pyy*. (***P < 0.001; **P < 0.01; *P < 0.05, Dunn to control test). (**g**) Schematic representation of human and mouse *PYY* gene structure, indicating the exons and introns and highlighting the presence of GC boxes in the first 100 bases of the promoter.

As these cell lines have low expression of *PYY* and may not respond in the same way as EECs *in vivo*, we used primary intestinal epithelial cultures to validate these results. Incubation of human colonic primary cultures with SCFAs also increased the expression of *PYY*: butyrate and propionate increased expression ~16-fold and ~7-fold respectively, whereas acetate increased expression ~2-fold. Again, this effect was specific to *PYY* and no significant stimulation of *GCG* expression was observed (Fig. 3a). The main effect of propionate and butyrate appeared to be due to their HDAC inhibitory activity as TSA reproduced a similar effect. Qualitatively similar effects were also seen in primary cultures from human small intestine, but the magnitude of the stimulation was smaller (Fig. 3b). Similar to the NCI-h716 cells, human primary cultures incubated with butyrate or TSA for 24 h secreted more PYY but not GLP-1 when subsequently incubated in the absence of acute secretory stimulants. Preincubation with butyrate or TSA further increased acute PYY, but not GLP-1-secretory responses to the previously reported strong secretory cocktail of glucose, forskolin and IBMX^7^, demonstrating that increased *PYY* mRNA expression translated into increased secretory capacity (Fig. 3c and d).

Interestingly, SCFAs had no effect on *Pyy* gene expression in mouse colonic primary cultures (Fig. 3e). TSA also had no effect on *Pyy* expression despite increasing the expression of other genes known to be regulated by HDAC inhibition at the same concentration (Fig. 3f), indicating that mouse *Pyy* expression may not be regulated by HDAC inhibition in contrast to the observed responsiveness of human *PYY*. This motivated us to compare the PYY gene structure between the two species. Whereas the human *PYY* gene includes 4 non-translated 5′ exons spanning 50,000 bases (Fig. 3g), the murine *Pyy* sequence comprises only one non-translated 5′ exon. None of the three additional human exons had homology with the 5′ region of the mouse *Pyy* gene. We could find three GC boxes in the first 100 bases of the human promoter, domains recognized by SP1/SP3, that have been implicated in butyrate HDAC inhibitory effects^16^. No GC-boxes were apparent in the mouse *Pyy* proximal promoter or the human *GCG* promoter, which may explain why the effect of butyrate was limited to human *PYY* expression.

## Discussion

Using human enteroendocrine cell lines, we characterized for the first time an important action of SCFAs, particularly propionate and butyrate, to increase *PYY* gene expression in human EECs. No changes were observed in transcription factors implicated in enteroendocrine cell number or differentiation, and the effect was specific to *PYY* but not *GCG*, although it should be noted that new EECs are not generated under our mixed primary culture conditions^36^ and that the human cell lines are a model of already EEC-committed cells^32^, leaving the possibility that *in vivo* increased differentiation of intestinal stem cells towards PYY and GLP-1 expressing cells might contribute to additional beneficial SCFA effects. The concentrations of SCFA we used are within the physiological range observed *in vivo* in the colon and are high enough to fully stimulate both human and murine FFA2 and FFA3. Pharmacological activation of FFA2, but not FFA3 or GPR109a, induced a small increase of *PYY* expression, which seemed insufficient to account for the strong stimulation induced by propionate or butyrate. Importantly, a strong stimulation was preserved in FFA2 depleted cells. HDAC inhibitors, by contrast, reproduced the strong effect of butyrate and propionate on *PYY* expression, and butyrate increased the expression of other genes known to be regulated by HDAC inhibition in these cells^17,37,38^, confirming its activity as an HDAC inhibitor in this model. We therefore concluded that HDAC inhibition likely explained the strong enhancement of *PYY* expression by propionate and butyrate. Interestingly, the strong stimulatory effect of SCFAs on *PYY* expression could be reproduced in human but not mouse primary intestinal cultures and TSA similarly only increased *PYY* expression in human tissues. We suggest that the difference between the two species reflects variation at the promoter level. The presence of three SP1/SP3 binding GC boxes, which have been shown to be important elements in HDAC-regulated gene expression downstream of butyrate^16^, likely explain the selective stimulation of human, but not murine *PYY*-expression.

The increased expression of *PYY* mRNA resulted in higher secretion of PYY peptide, both under basal and stimulated conditions. SCFAs are therefore likely to increase PYY plasma levels both in fasted and fed states through this mechanism, and the pathway might contribute to the known long term effects of fibre-rich diets^39^ or prebiotics^40^ on PYY but not GLP-1 plasma levels due to the increased SCFA production through fibre fermentation. The effect we observed was concentration dependent and can therefore be considered as a mechanism capable of sensing microbial fermentation activity. SCFAs are also an energy source for the body and the described pathway can be considered as a way for the host to sense long term energy availability and correspondingly to adapt energy intake. However, as the different SCFAs did not induce identical responses, the ratio of their production seems important. The relative production of different SCFAs is mainly controlled by the diet and the different types of fibre, as well as by the different bacterial species present in the gut microbiota. For example, obesity has been associated with a decreased number of butyrate and propionate producing bacterial species in favour of acetate producers^41^. Decreased gut concentrations of butyrate and propionate could therefore contribute to the decreased PYY levels seen in these patients through the mechanism presented here. Moreover, as PYY is known to regulate colonic motility and electrolyte secretion, modulating its biosynthesis and secretion may be an important way to regulate luminal contents and their concentrations. The pathway described here can be seen as a crosstalk between the gut microbiota and the host, in which bacterial metabolites regulate gut hormone levels that can in turn modulate the microbial environment.

## Material and Methods

### Cell culture and reagents

NCI-h716 cells (ATCC, CCL-251) and HuTu-80 cells (ATCC, HTB40) were maintained respectively in RPMI-1640 (Gibco, Life Technologies) and in EMEM (Sigma-Aldrich), all supplemented with 10% fetal bovine serum, 2 mM L-Glutamine, 50 IU/mL penicillin and 50 μg/mL streptomycin in a humidified incubator at 37 °C, 5% of CO2.

Primary cultures were prepared as already described^7,36^. Human tissues were obtained from Tissue Bank at Addenbrooke’s hospital, Cambridge, as approved by the local ethical review committee (09/H0308/24). All animal procedures were approved by the University of Cambridge Animal Welfare and Ethical Review Body and conformed to the Animals (Scientific Procedures) Act 1986 Amendment Regulations (SI 2012/3039). The work was performed under the UK Home Office Project License 70/7824. Mice were sacrificed by cervical dislocation and intestinal tissue used in the experiments. Cleaned tissues were digested with collagenase type XI (0.5 mg/mL for human tissue, 0.4 mg/mL for mouse tissue) and the crypt suspension plated into Matrigel coated plates and cultured for 24–48 h in DMEM 25 mM glucose supplemented with 10% FBS, 2mM L-Glutamine, penicillin, streptomycin and 1/1000 Y-27632 (Tocris).

Drugs and reagents were from Sigma-Aldrich. SCFAs, tiglic acid, 1-methylcyclopropane carboxylate (1-MCPC) and niacin were solubilized in water and pH adjusted between 7.2 and 7.4. Phorbol-12-myristate-13-acetate (PMA), Trichostatin-A (TSA), Forskolin and isobutyl-1-methylxanthine (IBMX) were solubilized in DMSO; final concentration of DMSO had no detectable effect on cells.

### Plasmid construction and transfection

Cloning of FFA2 was performed by PCR amplification from NCI-h716 genomic DNA and integrated after EcoRI and XhoI digestion into a pCMV-eGFP-N1 vector (a kind gift from A. Echard, Institut Pasteur, Paris). Oligonucleotides used for amplification of *FFAR2* were 5′-*aaaactcgagatgctgccggactggaa*-3′ and 5′-*aaaagaattcctactctgtagtgaagtccga*-3′. HuTu-80 cells were seeded at 2 × 10^5^ cells per well in 6-well plates 24 h before transfection. For transfection, medium was removed and replaced with Optimem (Gibco, Life Technologies) and 2 μg of plasmid DNA mixed with 4 μL lipofectamine 2000 (Invitrogen) in 50 μL Optimen were added following manufacturer’s instructions. Medium was replaced 6 h after transfection, and experiments were performed 24 to 48 h after transfection.

### FFA2 depletion

Crispr/Cas9 was designed to specifically target *FFAR2* coding sequence about 100 bases downstream of the start codon. Guide RNA (TGGCCCTGCGGGCCTTTGTGGGG) was integrated in a pX330-U6-Chimeric_BB-CBh-hSpCas9 vector containing guideRNA and Cas9 sequence, following published protocols (a gift from Feng Zhang, Addgene plasmid #42230)^42^. Clonal populations of transfected NCI-h716 cells were selected based on their absence of response to SCFAs in calcium imaging, and gene disruption was confirmed by sequencing the region surrounding the targeted sequence by the RNA guide using the same oligonucleotide as the ones used for *FFAR2* cloning. *FFAR3* sequence and expression was confirmed to remain unaltered. The oligonucleotide to amplify *FFAR3* sequence were ATGGATACAGGCCCCGACCAG and CTAGCTTTCAGCACAGGCCAC, and sequencing was performed using nucleotides allowing a complete covering of the gene (CAGGCGGCTGTAGCAGTAGC and GGCCGAGGCTGGGGCAGGCA).

### RNA extraction and qPCR

NCI-h716 (1 × 10^6^ in 12-well plates) or HuTu-80 (5 × 10^5^ in 6-well plates) cells were seeded 48 hours before lysis. Drugs were added 24 h before RNA extraction if not indicated otherwise. RNA was extracted using an RNeasy minikit with DNAse treatment (Qiagen). 2 μg of RNA were used for reverse transcription using High capacity cDNA Reverse transcription kit (Applied Biosystems). qPCRs were performed in duplicates on an AbiPrism 7000 system with Taqman gene expression assay probes (Supplementary Table S1) and Taqman gene expression master mix (LifeTechnologies). Data were analyzed using a 7000 System SDS software (Applied Biosystems) and represented as the mean + -s.e.m. of the fold change of expression of at least three different experiments. Statistical analysis was performed on the fold change, using the Dunn to control test.

### Secretion studies

For secretion studies, 4 × 10^5^ NCI-h716 cells per condition were centrifuged and rinsed twice with saline buffer (NaCl 140 mM, KCl 5 mM, MgCl_2_ 2 mM, CaCl_2_ 2 mM, Hepes 10 mM, pH adjusted to 7.3). Human primary culture were rinsed twice with saline buffer. Cells were incubated 2 h in 200 μL of saline buffer with DiprotinA (30 mM) at 37 °C. Supernatant was centrifuged once and stored at −80 °C until used for ELISA. PYY and GLP-1 concentration in the supernatant of cell lines was measured using a total PYY and a total GLP-1 Elisa kit (EZHPYYT66K and EZGLP1T-36K, Merck Millipore) using manufacturers’ instructions. Measurements were performed with a microplate reader (Infinite 2000, Tecan). GLP-1 and PYY measurement from primary cultures were performed by the Core Biochemical Assay Laboratory, Cambridge, UK using total GLP-1 and total PYY assays (MesoScale Discovery (MSD), Gaithersburg, MD, USA) and normalized to the total protein content of the lysed culture. Data was analyzed using R, presented as the fold change relative to the Control incubation/Control stimulus and statistical analysis was assayed using the Dunn to control test, testing the effect of the incubation independently to the secretion stimulus.

### Data availability

No datasets were generated or analysed during the current study. Raw data and specific materials are available by contacting the corresponding author.

## Electronic supplementary material

Supplementary information
