# Supplementary material for: SCFAs strongly stimulate PYY production in human enteroendocrine cells
Source: Sci Rep. 2018 Jan 8;8:74. doi: 10.1038/s41598-017-18259-0 (PMC5758799; doi:10.1038/s41598-017-18259-0)

SCFAs strongly stimulate PYY production in human enteroendocrine cells

P. Larraufie, C. Martin-Gallausiaux, N. Lapaque, J. Dore, F.M. Gribble, F. Reimann, H.M. Blottiere

Supplementary information

Material and method

Calcium imaging

Clonal cell lines stably expressing GCaMP-5G (a gift from D. Kim and L. Looger (Addgene plasmid #31788)^1^) were selected by resistance to G418 (500μg/mL). NCI-h716 GCaMP-5G cells were coated in a 96-well plate with μclear bottom for microscopy (Greiner BioOne) few hours before experiments in saline buffer (NaCl 140mM, KCl 5mM, MgCl_2_ 2mM, CaCl_2_ 2mM, Hepes 10mM, sucrose 6mM and glucose 10mM, pH adjusted to 7.35). Cells were observed on a Zeiss LSM700 confocal laser scanning microscope (MIMA2 platform, INRA) at 25°C and filmed for 4 minutes at 1 image per second. Reagents were added 50s after beginning of acquisition. Image analysis and cell fluorescence were done using Fiji software^2^ and data analysis was performed using R software. Data was analyzed as the relative fluorescence to mean fluorescence before reagent addition.

Figure legend

Supplementary figure 1

A: chromatograms showing the sequence targeted by the CRISP described in material and methods, in wild type cells (top) and in the clonal population selected for its depletion of active FFAR2 (bottom). B: Functional validation of the depletion of FFAR2 in the ko cells by calcium imaging: Acetate induce a calcium response in NCI-h716 cells due to FFAR2 stimulation^3^ (left) whereas the response was abolished in the ko FFAR2 cells. C: Expression of FFAR2 and FFAR3 in WT and FFAR2 ko mice, measured by qRT-PCR.

Supplementary references

1. Akerboom, J. *et al.* Optimization of a GCaMP calcium indicator for neural activity imaging. *J. Neurosci.* **32,** 13819–40 (2012).

2. Schindelin, J. *et al.* Fiji: an open-source platform for biological-image analysis. *Nat. Methods* **9,** 676–82 (2012).

**Supplementary table 1**

| GAPDH | Hs02758991_g1 | FFAR2 | Hs00271142_s1 |
| --- | --- | --- | --- |
| CYCLOPHILLIN A | Hs04194521_s1 | GPR109a | Hs02341584_s1 |
| B-ACTIN | Hs01060665_g1 | NEUROGENIN3 | Hs01875204_s1 |
| PYY | Hs00373890_g1 | FOXA1 | Hs04187555_m1 |
| GCG | Hs01031536_m1 | PAX6 | Hs00240871_m1 |
| CHROMOGRANIN A | Hs00900375_m1 | PAX4 | Hs00173014_m1 |
| MCT1 | Hs00161826_m1 | B-act (mouse) | Mm02619580_g1 |
| SLC5A8 | Hs00377618_m1 | Pyy (mouse) | Mm00520716_g1 |
| FFAR3 | Hs02519193_g1 |  |  |


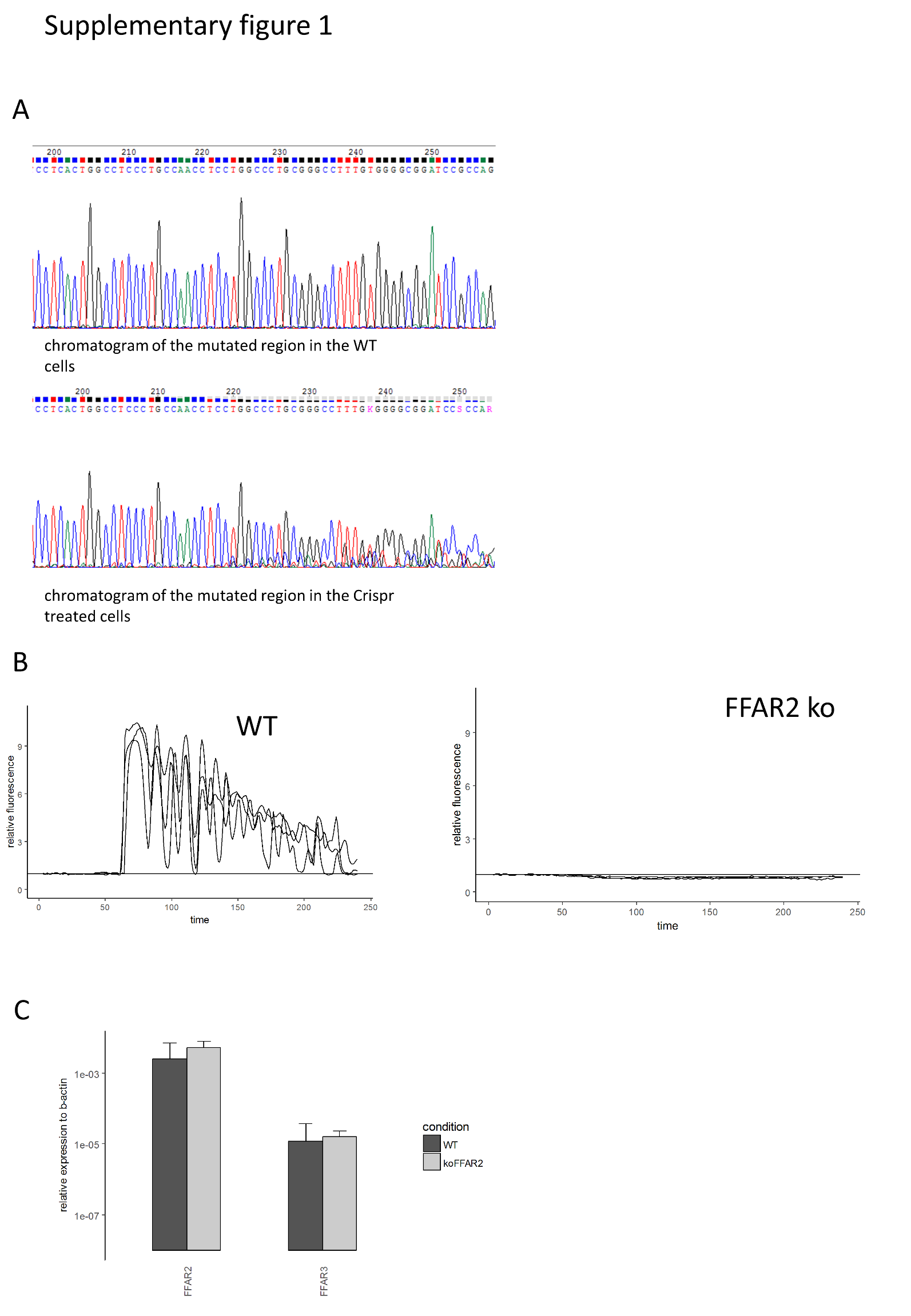

Supplement: Supplementary file 1 — Supplementary information [file 41598_2017_18259_MOESM1_ESM.doc]
